# Mild Brain Injury

**DOI:** 10.1155/2012/469475

**Published:** 2012-10-13

**Authors:** Brian D. Greenwald, Anne Felicia Ambrose, Gina P. Armstrong

**Affiliations:** ^1^Department of Rehabilitation Medicine, Mount Sinai School of Medicine, New York, NY 10029, USA; ^2^Department of Physical Medicine & Rehabilitation, UT Medical Center, Dallas, TX 77030, USA

Mild traumatic brain injury (mTBI), commonly known as concussion, is one of the most common neurologic disorders [1]. Based on emergency room visits, hospitalizations, and deaths, an estimated 1.7 million people sustain a traumatic brain injury (TBI) annually in the United States [2]. About 80% of TBIs that occur each year are mild TBI [3]. Since many people are not evaluated in a medical setting, the true annual incidence of mTBI is thought to be much higher. Estimates of the incidence of sport-related mTBI range from 1.6–3.8 million per year in the United States [4]. Research indicates that up to 15% of persons who suffer an mTBI may have persistent disabling problems [5, 6].

Despite the high incidence, mTBI has only entered the consciousness of the medical community and lay press in the last decade. This interest has been fueled by combat-related injuries as well as high-profile athletes. There has been increased public awareness regarding the need for evaluation and treatment of mTBI as well as possible long-term health effects. Chronic traumatic encephalopathy (CTE) is a neurodegenerative disease thought to be caused, at least in part, by repetitive brain trauma that can occur during contact sports and military participation [7, 8]. CTE has also become a popular topic due to its close association with sports like American football, hockey, soccer, boxing, and professional wrestling. Only small uncontrolled studies currently exist regarding the prevalence, risk factors, and natural history of CTE.

Recent reports in the lay press about professional athletes, in particular in the National Football League, have led to fears about the cumulative effects of mTBI on children, adolescents, and college athletes and risk of CTE. State governments have created laws in response to these fears. As of May 2012, 38 states (plus the District of Columbia and the city of Chicago) had adopted youth concussion laws. The main tenets of the laws include the following: inform and educate youth athletes, their parents, and guardians and require them to sign a concussion information form; removal of a youth athlete who appears to have suffered a concussion from play or practice at the time of the suspected concussion; require a youth athlete to be cleared by a licensed health care professional trained in evaluation and management of concussions before returning to play or practice. To promote the prevention of, recognition of, and appropriate responses to TBI, the CDC has developed the Heads Up initiative, a program that provides concussion and mild TBI education to specific audiences such as healthcare providers, coaches, athletic trainers, school nurses, teachers, counselors, parents, and student athletes [9].

The Centers for Disease Control and the World Health Organization have promoted that the term “concussion” be replaced by the term “mild traumatic brain injury.” There is ongoing debate over whether mTBI is synonymous with concussion or not. mTBI and concussion have been used interchangeably, although the latter is more commonly used in sports and mTBI in general medical contexts [10, 11].

In comparison with moderate and severe traumatic brain injuries, mTBIs are often more challenging to diagnose. Often there is swift resolution of signs and symptoms and typical absence of evidence of injury on standard neuroimaging. Because of this, in 1993 The American Congress of Rehabilitation Medicine (ACRM) (Mild Traumatic Brain Injury Committee, 1993) was the first organized interdisciplinary group to advocate for specific criteria for the diagnosis of a mild TBI (Table 1) [12]. This ACRM definition has been widely used, especially in the field of rehabilitation and neuropsychology. Centers for Disease Control (CDC) and the World Health Organization (WHO) have advanced a standardized definition that was derived from the ACRM definition [13, 14].

Recently an expert consensus relating to soldiers returning from Iraq and Afghanistan used the same clinical signs to define mTBI [15]. For these soldiers, the most frequent combat-related injury incurred is TBI, in particular mTBI [15]. Approximately 22% of the wounded soldiers arriving at Landstuhl Regional Medical Center in Germany have head, neck, or face injuries. TBIs result primarily from improvised explosive devices (IEDs), landmines, high-pressure waves from blasts, blunt force injury to the head from objects in motion, and motor vehicle accidents [16, 17]. mTBI characterizes most of the blast-induced traumatic brain injuries seen in service members returning from Iraq and Afghanistan, with reports of 300,000 service members sustaining at least one mTBI as of 2008 [18]. The frequency of these injuries has led to mTBI being called the “signature injury of war” [16].

mTBI is a major health problem that affects both civilians and military populations. This edition of the journal Rehabilitation, Research and Practice reviews the up-to-date literature on the diagnosis, management, and outcomes of MTBI in both populations. Screening for mTBI and return to play after sports concussion are also reviewed. It is our desire that readers gain further insight into the complexities of this disorder, useful tools for diagnosis, and further treatment options.

*Brian D. Greenwald* *Brian D. Greenwald* *Anne Felicia Ambrose* *Anne Felicia Ambrose* *Gina P. Armstrong* *Gina P. Armstrong*

## Figures and Tables

**Table 1:** Diagnostic criteria (Mild Traumatic Brain Injury Committee, Head Injury Interdisciplinary Special Interest Group, American Congress of Rehabilitation Medicine) [12].

A traumatically induced physiological disruption of brain function, as manifested by at least one of the following	
Any loss of consciousness	
Any loss of memory for events immediately before or after the accident	
Any alteration in mental state at the time of the accident (e.g., feeling dazed, disoriented, or confused)	
Focal neurologic deficit(s) that may or may not be transient	
But where the severity of the injury does not exceed the following	
loss of consciousness of approximately 30 min or less	
after 30 min, an initial Glasgow Coma Scale score of 13–15 and	
post-traumatic amnesia not greater than 24 hr	
